# Leveraging whole blood based functional flow cytometry assays to open new perspectives for rheumatoid arthritis translational research

**DOI:** 10.1038/s41598-022-16622-4

**Published:** 2022-07-16

**Authors:** Celia Cartagena García, Nathalie Balandraud, Jean Roudier, Pierre Lafforgue, Nathalie Lambert, Jean-Marc Busnel

**Affiliations:** 1 Research Department, Beckman Coulter Life Sciences, Marseille, France; 2 INSERM UMRs 1097, Aix Marseille University, Marseille, France; 3 AP-HM, Rheumatology, Sainte Marguerite Hospital, 13014 Marseille, France

**Keywords:** Biological techniques, Immunology, Biomarkers, Medical research, Rheumatology

## Abstract

Despite introduction of biological disease modifying anti-rheumatic drugs (DMARDs) for Rheumatoid arthritis (RA) treatment, therapeutic strategies do not always lead to disease control and remission. Hence, a more efficient patient stratification and monitoring biomarkers and tools are needed to enable a more personalized medicine. We used a whole blood based functional flow cytometry assay to characterize immune cells from RA patients (treated or not), healthy donors and psoriatic arthritis (PsA) patients according to their responses to LPS and/or anti-TNFα (infliximab, IFX). Activation marker expression was measured using a 10-color flow cytometry panel following a no-wash protocol. Naïve-to-treatment RA patients had a stronger inflammatory profile in comparison to healthy donors at basal level. Higher expression of activation markers (CD69 and/or CD11b) on NK, B cells and granulocytes and lower expression of the adhesion molecule CD62L were measured on monocytes, granulocytes and B cells. After LPS, naïve RA patients’ cells were less capable of regulating CD69, CD11b, CD16 or CD62L showing impaired activation capabilities. Upon LPS and IFX co-incubation, hierarchical clustering analysis showed different profiles between cohorts. We believe that this whole blood-based approach should further be assessed for RA patient characterization as it provides new perspectives for stratification and/or monitoring.

## Introduction

Rheumatoid arthritis (RA) is an autoimmune disease that causes pain, stiffness and joint destruction. According to the European League Against Rheumatism (EULAR), this condition affects between 0.3 and 1.0 % of the general population. Rheumatoid factors or ACPAs (anti-citrullinated protein antibodies) are commonly used nowadays in the diagnosis of RA, specially ACPAs since they are RA-specific and present in 70-80% of cases^1,2^.

Pro-inflammatory mediators such as eicosanoids or cytokines contribute to RA inflammation, initiation and sustainment^3,4^. Tumor Necrosis Factor-α (TNF-α) is one of the strongest contributors to inflammation chronicity and acts through two different signalling pathways called forward (FS) and reverse signalling (RS). In brief, FS has a pro-inflammatory outcome leading to proliferation, anti-apoptosis and inflammation, while RS triggers anti-inflammatory and apoptotic responses^5^.

Therapeutic options have been increasing over the past decades to yield several possible strategies for RA treatment^6^. Conventional disease modifying anti-rheumatic drugs (DMARDs) such as methotrexate have been historically used as a first line therapy^7^. Second lines biological DMARDs are now added to reach disease remission. Through blocking FS, triggering RS and/or antibody dependent cell cytotoxicity (ADCC), anti-TNFs represent an important innovation in the field and have commonly been used since their introduction^8^.

RA patients are monitored after treatment prescription to follow disease evolution and assess therapeutic efficiency. It is usually considered that each treatment should be implemented for at least 3 months before observing possible improvement on patients and 6 months to achieve therapy target before therapeutic strategy adjustment^9^. Unfortunately, disease heterogeneity, causing differences in donor characteristics, leads to diverse patient’ response to therapy, thus not all patients achieve disease control. As a whole, this process of adapting treatment and identifying the right therapeutic strategy for a given patient is lengthy and may significantly delay the achievement of disease remission. It may also lead to unresolvable medical complications. Hence, biomarkers and tools that could help predict response to treatment and improve current therapeutic approaches are strongly desired.

In our previous study^10^, using samples from healthy individuals, we developed a whole blood–based functional assay strategy to characterize and assess anti-TNFs mechanisms of action (MOAs) within a donor’s referential. As this primary objective was pursued, the intent was also to provide information relevant to the study of inflammatory conditions in general. As a result, markers either capable of informing on MOAs or known to be of interest and/or dysregulated in RA conditions were combined^11–21^. We thus designed a 10-color flow cytometry panel where, apart from gating markers, 6 activation markers were considered. CD69 was used to assess the general activation status of cells, adhesion molecules such as CD11b (ITGAM), CD54 (ICAM-1), CD62L (L-selectin), or CD66b (CEACAM8) were included to study migration/adhesion processes. An anti-TNF fluorochrome conjugated antibody was also used to study the expression of transmembrane TNF upon in-vitro treatment with anti-TNF therapeutics. . Finally, CD16, which may serve as a gating or an activation marker was used to inform on the potential engagement of NK cells through the Antibody Dependent Cell Mediated Cytotoxicity (ADCC) MOA.

Herein, we aimed to evaluate the relevance of the previously developed whole blood based functional assay for RA patient’ characterization and further stratification. To this end, whole blood samples from healthy individuals or RA patients, either naïve to therapy or already treated, were considered. Samples from psoriatic arthritis (PsA) patients were also included as an additional set of patients suffering from an inflammatory disease to further test the relevance of the proposed approach. By stimulating cells with LPS, or LPS in combination with IFX, we hypothesized that besides basal immune cell marker expression, LPS stimulation capacity and further response to IFX could provide valuable insights and serve as possible biomarkers in the future to stratify patients and/or predict response to therapy.

## Materials and methods

### Samples

Upon given consent according to Helsinki declaration, blood from 21 healthy donors were collected from volunteers visiting St. Joseph Hospital (Marseille, France). Samples from 33 RA (16 naïve,17 treated) patients fulfilling ACR/EULAR 2010 criteria and 13 psoriatic arthritis (PsA) patients fulfilling CASPAR criteria were collected at the Sainte-Marguerite (AP-HM) hospital (Marseille, France). Patient characteristics are detailed in Supplementary Table 1. Clinical scores (DAS28 for RA patients and DAPSA for PsA patients) are depicted in Supplementary Table 2.

### Whole blood based functional assay—flow cytometry

The approach implemented here was similar to the one already described in our first proof of concept study in which the assay was initially developed and characterized from different standpoints, including mechanistic relevance and repeatability (Cartagena Garcia *et al*, 2020). Heparinized whole blood was used to avoid metal ion sequestration. In brief, 50µL of whole blood were incubated per condition, including a negative control, an LPS (200 ng/mL) (Sigma Aldrich) control and an LPS+IFX (1 µg/50µL whole blood) (Merck) combination. After 5 h of incubation at 37 °C, cells were stained for 30 min at room temperature and protected from light with the following staining mix: CD3-allophycocyanin (APC) Alexa Fluor® (AF) 750 (UCHT1), CD11b-PE (Bear1), CD14-Pacific Blue (RMO52), CD16-ECD (3G8), CD45-KrO (J33), CD54-FITC (84H10), CD56-PC5.5 (N901), CD62L-APC (DREG56), CD66b-APCAF750 (80H3), CD69-PC7 (TP1.55.3), and tmTNF-AF700 (HRS4) (all from Beckman Coulter Life Sciences). A centrifuge-free protocol was then followed to simplify workflow. Red blood cells were lysed using OptiLyse C following manufacturer instructions for use (Beckman Coulter Life Sciences). Finally, marker expressions were measured with a three-laser 13-color CytoFLEX flow cytometer (Beckman Coulter Life Sciences) within 5 hours of staining. Gating strategy used for flow cytometry analysis is displayed in Supplementary Figure 1.

### Statistical analysis

Flow cytometry data were analyzed with Kaluza Analysis Software version 2.1 (Beckman Coulter). Populations of interest were manually gated and JMP 14.2.0 (SAS Institute) was used for uni/multivariate statistical analysis. Raw or normalized Mean Fluorescent Intensity (MFI) values were considered depending on the condition studied. Raw MFIs were used to study basal marker expression levels while LPS or LPS+IFX conditions were normalized by the negative or LPS conditions respectively, thereby obtaining stimulation indexes (Stim. Index). Unsupervised analysis using JMP’s response screening platform was used to identify most discriminative parameters based on t-student or ANOVA test results and false discovery rate (FDR) corrected p-values. Principal component analyses (PCA) were carried out considering most discriminative features. To further study divergences between cohorts, box plot representations were used and hierarchical clustering was conducted. Differences in MFI and/or stimulation indexes were analyzed by a non-parametric Wilcoxon test for which *p-values inferior to 0.05 were considered significant.

### Ethics declarations

All enrolled patients provided informed consent and procedures followed were in accordance with the Helsinki Declaration. Care of the subjects was not modified, and the results of the study had no influence on subjects’ management. Healthy whole blood samples were from donors receiving general work-up at the Hospital Saint Joseph (Marseille, France). Under the French law, ethics committee approval and donor consent were not required for this type of non-interventional study, provided the donors had received information and retained the right to oppose the use of excess sample and anonymized medical data (Loi n°2012–300 du 5 mars, 2012). Regarding patient samples, whole blood from sixteen RA naïve, 17 treated and 13 PsA patients weas used and all considered patients gave informed written consent for this study. Patient sample collection was approved by the Committee of Protection of Persons (CPP) Sud-Méditerranée II (committee reference: 208 C10) and the services of the Direction Générale de la Recherche et de l’Innovation du Ministère under the identification number DC-2008-327. Cell analyses were performed on pseudonymized samples and all data collected in the study were retrieved from subject records by the practitioner.

## Results

### CD69, CD11b and CD62L basal expressions of some cell subsets differed between healthy and naïve RA patients

**Figure 1:**
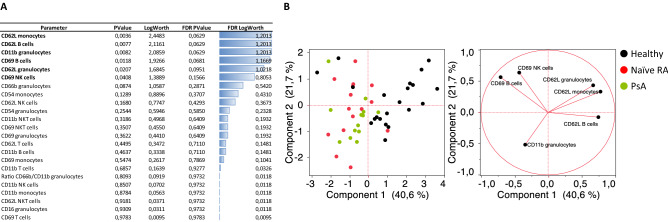
Most discriminative basal immunophenotypic markers between healthy donors, naïve to treatment RA and PsA patients. (**A**) Response screen of basal Mean Fluorescent Intensities (MFIs) ranking all parameters according to their ability to discriminate healthy from naïve RA patients. (**B**) Principal component analysis considering most discriminative features only (p-value < 0.05).

An unsupervised analysis was first conducted to assess whether the basal expression of the activation markers considered could represent discriminative features between healthy and naïve RA patients (Fig. 1A). CD62L expression on monocytes and B cells as well as CD11b on granulocytes came up as the most discriminative features with p-values below 0.01. CD69 on B cells and NK cells together with CD62L on granulocytes also had significant p-values. A Principal Component Analysis (PCA) was then performed selecting the 6 most discriminative parameters with p-values below 0.05 (Fig 1B). Naïve and healthy donors clustered separately except 3/21 healthy donors and 1/16 naïve patient. Control PsA patients mostly overlapped with naïve RA patients.

**Figure 2:**
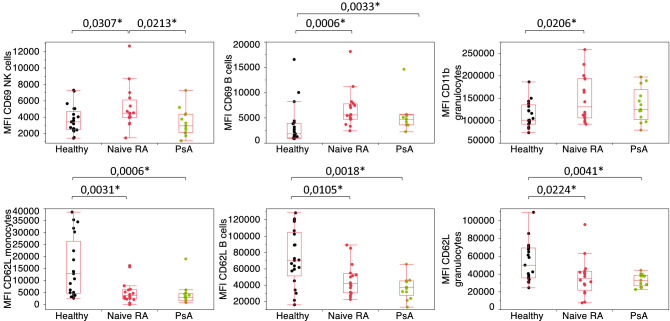
Most discriminative immune marker basal expression in healthy, naïve RA and PsA patients. CD69 Mean Fluorescent Intensity (MFI) expression in NK and B cells, CD11b MFIs in granulocytes, CD62L MFI in monocytes, B cells and granulocytes. Statistical significance was determined by a nonparametric Wilcoxon test, with *p < 0.05.

To further investigate whether these parameters were up or downregulated in RA patients, box plots were represented (Fig. 2). Activation markers such as CD69 on several subsets and CD11b on granulocytes were significantly elevated in naïve RA patients in comparison with healthy controls. Conversely, naïve RA patients exhibited downregulated CD62L on monocytes, B cells and granulocytes. While some marker expressions appeared to be similar between naïve RA and PsA patients, others such as CD69 on B cells, CD62L and CD69 on NK cells or CD11b on granulocytes significantly differed between the two inflammatory disease subgroups (Supplementary Figure 2).

### Cellular stimulation capabilities were lower in RA patients according to the expression of several activation markers

**Figure 3:**
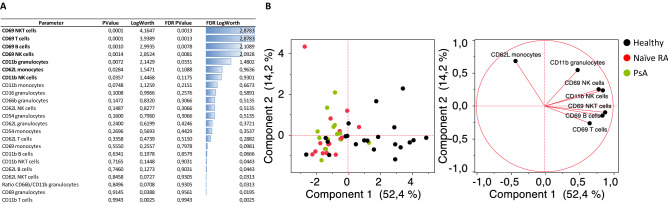
Multivariate analysis of all parameters considered for the LPS condition from heathy donors, naïve RA and PsA patients. (**A**) Response screen of Stimulation indexes (Stim. Index) ranking all parameters according to its discriminative power to distinguish healthy from naïve RA patients after LPS stimulation. (**B**) Principal component analysis were only most discriminant features having a p-value < 0.05 were included in the analysis.

After comparing basal activation marker expression levels, we then investigated the immune cell stimulation capabilities of healthy controls and RA patients following the approach described beforehand. In Fig. 3, results of an unsupervised analysis discerning most discriminative parameters between groups are shown. As showed in our previous study (Cartagena Garcia et al, 2020), not only monocytes but all major immune cell subsets were stimulated upon LPS treatment as a result of the cascade reaction initiated by the effect of LPS on monocytes. Under these activation conditions, CD69 in NKT, T, NK, and B cells were identified as the top discriminators between healthy and naïve RA patients. Adhesion molecules such as CD11b on granulocytes and CD62L on monocytes, also had p-values lower than 0.05. In spite of some distinct spatial separation between cohorts, no evident clustering was observed when considering the 7 best discriminators for a subsequent PCA analysis (Fig. 3B).

**Figure 4:**
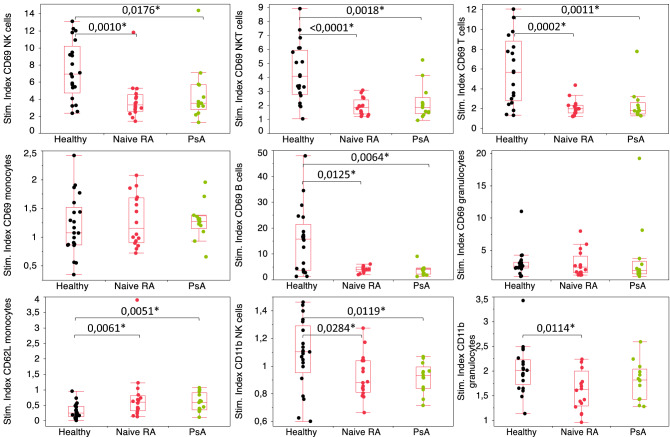
Box plots for CD69, CD62L and CD11b expression after LPS stimulation in healthy, naïve RA and PsA patients. Stimulation indexes (Stim. Index) were obtained by normalizing MFI values from the LPS condition by the negative condition. Statistical significance was determined by a nonparametric Wilcoxon test, with *p values < 0.05.

As one of the most discriminative markers in several subsets, we then analyzed in more details the general activation marker CD69. As shown in Fig. 4, lower values of stimulation indexes were found in RA and PsA patients in comparison to healthy controls. This decrease was visible on several cell types, including NK, NKT, T and B cells. In contrast, no differences were found for monocytes nor granulocytes. Not only CD69 but also CD11b and CD62L stimulation indexes differed between cohorts. While CD62L had a similar behavior in RA and PsA patients, with less downregulation upon LPS stimulation than in healthy volunteers, RA patients presented significantly larger downregulations of CD11b on NK cells and granulocytes than both healthy controls and PsA samples.

### In vitro response to infliximab differed between healthy, naïve and treated RA patients

**Figure 5:**
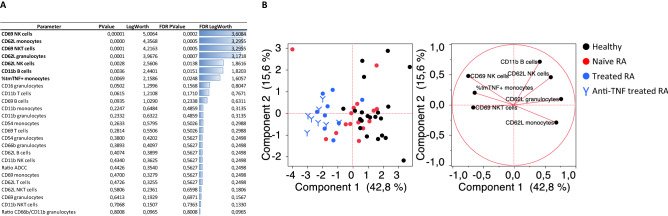
Multivariate analysis of all parameters considered for the LPS+IFX condition. healthy donors, naïve and treated RA patients in the LPS+IFX condition. (**A**) Response screen of Stimulation Indexes of immune cell markers. Stimulation Indexes were obtained by normalizing LPS+IFX MFI values by LPS MFIs. (**B**) Principal component analysis were only most discriminant features having a p < 0.05 were included in the analysis.

Since response to treatment remains one of the biggest challenges in RA management, we next wondered about the *ex vivo* capability to respond to a well-known anti-TNF (infliximab) to assess whether patients’ stratification and monitoring could potentially benefit from such approach. Unsupervised analysis was performed considering the healthy, naïve RA and treated RA patient cohorts (Fig. 5A). As previously, we adopted an unsupervised analysis approach subsequently conducting a response screen and a PCA analysis (Fig. 5B). CD69 on NK and NKT cells, CD62L on NK cells, monocytes and granulocytes, CD11b on B cells and percentage of tmTNF^+^ monocytes were found to be discriminative features (p-values < 0.05), and considered for PCA analysis. While healthy donors and naïve RA patients overlapped on the PCA plot, treated RA patients, no matter the treatment, were significantly separated from the rest of individuals, forming a rather well-defined cluster with only 1 treated RA patient out of 17 not included.

**Figure 6:**
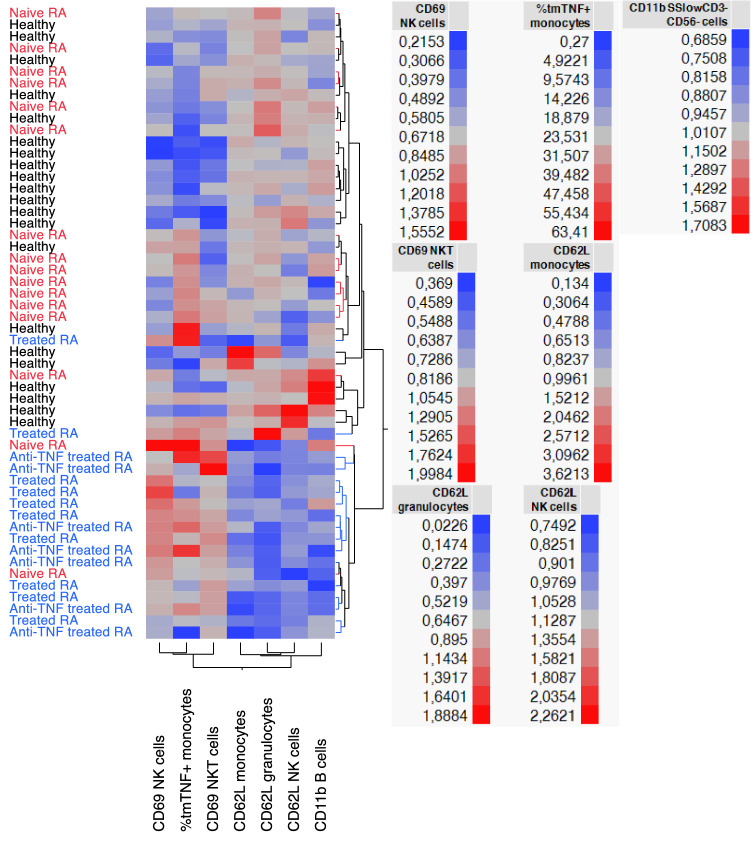
Dendogram based on two-way clustering analysis according to most discriminative parameters between healthy, naïve and RA treated patients. Unsupervised hierarchical clustering was performed considering healthy (black) naïve (red) and treated or anti-TNF treated patients (blue) RA patients where each row represents one donor. Legend is displayed for each parameter on the figure side.

Deepening more into the *in vitro* response to IFX, a hierarchical clustering analysis was carried out to investigate in details differences existing between groups (Fig. 6). Of note, CD62L stimulation indexes in NK cells, monocytes and granulocytes were lower in RA treated patients in comparison with naïve and healthy donors, suggesting a less potent effect of IFX in this cohort. Interestingly, CD11b on B cells showed a similar profile than CD62L. Contrarily, CD69 on NK, NKT and B cells as well as proportions of tmTNF^+^ monocytes were higher in treated patients. In addition to general trends, such analysis thus enabled to capture response heterogeneity between individuals.

**Figure 7:**
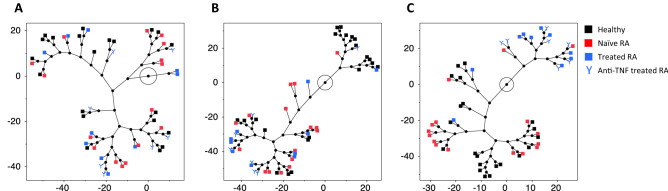
Sample clustering considering healthy donors, naïve and treated RA patients. Non-normalized negative control (**A**), LPS normalized condition by the negative control (**B**), LPS+IFX normalized by the LPS condition (**C**). Colors represent healthy (black) naïve (red) or treated RA patients. Each row is displayed by an end point, and each cluster join is represented by a new point. Lines represent distance between clusters and are relative to each other.

Interestingly, it was found that the donor clustering profile strongly depended on activation condition (Fig. 7). Using constellation plots, no obvious donor clustering could be found in the negative (Fig. 7A) nor LPS (Fig. 7B) conditions while the RA treated patients’ cohort generally clustered separately when infliximab was added to the mix (Fig. 7C).

## Discussion

Rheumatoid arthritis is a condition with heterogeneous patients’ characteristics for which treatment landscape has evolved significantly over the last decades. In addition to the historically used methotrexate, many biological therapies have been developed, and combination therapies have been included since EULAR 2013 updated recommendations for RA treatment^22^. Difficult-to-treat patients however remain an unsolved challenge that hinders management of this disease globally. Some reports propose that the non-responder rate for anti-TNF treatments can be up to 30-40%^23^. With such a high rate of patients not responding to therapy, there is a need for a better comprehension of immune characteristics, including biomarkers that could help improve therapeutic strategies.

RA has been extensively investigated using a variety of *in vivo* and *in vitro* pre-clinical models. Mice have commonly been used to perform fundamental studies. Other systems such as cell lines, human-derived cell like isolated cells or peripheral blood mononuclear cells (PBMCs) have also been explored to better reflect the human in vivo situation. Humanized mice also tend to be more frequently used nowadays, they however remain very expensive and have numerous limitations^24^. In the quest toward the development of new models in which human physiology would be even better represented^25^, new approaches have emerged and have been improved over the last decade, especially around the concepts of organoids and/or organ-on-a-chip^26^. 3D tissue engineering approaches that mimic structural features of the joint or microfluidic approaches have for example been recently discussed^27,28^. On top of being readily available, whole blood presents a multitude of advantages. If freshly drawn (24 h), it doesn’t only contain live immune cells whose functions can be interrogated, but also additional blood compartments, including but not limited to red blood cells, platelets, metabolites, lipids, proteins, antibodies, enabling complex pathways and/or interactions to happen. Here we hypothesized that whole blood based functional assays could significantly resemble *in vivo* human physiology and open valuable translational research perspectives. Moreover, by using a protocol in which sample manipulation is reduced to a minimum, we aim to provide a streamlined and broadly applicable workflow with many sources of variability removed.

We first investigated basal marker expression to highlight differences in activation marker levels between healthy donors and RA naïve patients as summarized in Supplementary Figure 3. As illustrated in Figs. 1 and 2, significant differences were found with CD69 upregulated on NK cells, B cells and granulocytes from RA naïve patients, the latest in accordance with Capsoni and colleagues^13^. Higher CD69 expressions in both NK and granulocyte compartments further confirm that innate immunity plays a central role in RA pathogenesis^29^. Proportions and activation status of synovial fluid NK cells have for example been shown to be higher in patients with advanced and active RA suggesting an important role in disease activity^30^. Neutrophil mechanisms of activation such as NETosis, oxidative stress and/or migration capabilities have also been reported to be dysregulated in RA and could significantly contribute to the disease^31,32^. The fact that RA patients express more CD69 on B cells could be related to a higher basal activation of B cells and to a higher immunoglobulin and auto-antibody production^33^.

In our settings, RA patients showed, in comparison to healthy donors, altered expressions of CD11b on granulocytes and of CD62L on monocytes, B cells and granulocytes. This is in agreement with previous studies that also reported such alterations in samples from RA patients^14–18^. CD11b and CD62L, also known as ITGAM and L-selectin, are adhesion molecules and have key roles in cell migration and chemotaxis processes. CD62L role in inflammation has already been outlined^34,35^ as being crucial for cell homing into tissues. CD11b is important for leukocyte rolling, adhesion, crawling and transmigration across blood vessels^36^. It can be hypothesized that the expression alteration of these molecules may contribute to higher cell infiltration rate into the synovial membrane.

Some of these characteristic features were also visible in the PsA cohort. CD69 on NK cells or CD11b and CD62L on granulocytes appeared to discriminate the RA group only, pointing again out toward a possibly major role of innate immunity in RA. The specific role of CD69 in RA pathogenesis has been already highlighted in different experimental models as required for the inflammatory cell response^11–13^. When comparing basal expressions of markers between the RA and PsA cohorts, a significant contrast was highlighted by PCA analysis (in Suppl Fig. S3) with CD69 on NK and NKT cells as well as CD62L on T cells being as the stronger discriminators.

We next investigated cell activation capabilities leveraging an *in vitro* LPS stimulation. Through TLR4 interaction, monocytes are initially activated and produce a variety of pro-inflammatory mediators, including TNF, causing a downstream activation cascade that induces a general immune cell activation^10^, thereby recreating an inflammatory environment. Despite an incomplete PCA segregation between cohorts, RA sample stimulation indexes after LPS stimulation were found to be significantly lower in a variety of subsets according to CD69, CD11b or CD62L expression. In view of these results, we envision two possible hypotheses. On the one hand, we confirmed that RA patients’ basal activation state is higher than the one of heathy donors, which might explain why the cell stimulation capabilities are lower in these patients. On the other hand, cells might be exhausted as a consequence of the chronic inflammation status existing in RA. Supporting this hypothesis, some exhaustion markers have been found to be higher in RA patients. For example, PD-1, either as a soluble form or on T cells, Tim-3 on peripheral NKT, T cells and monocytes^37–39^ as well as CTLA-4 on synovial T cells and Tregs^40,41^ were found to be dysregulated.

Finally, we studied the *in vitro* response to LPS and infliximab IFX to assess the ability of each individual/cohort to respond to this anti-TNF, including treated RA patients. Different clustering patterns were found, suggesting a different capability to respond to IFX. Stimulation indexes of CD69 on NK, NKT, B cells and T cells as well as of CD11b on granulocytes, were lower in treated RA patients, all informing about the forward signaling neutralization mechanism of action (MOA). Regarding the reverse signaling MOA, no significant differences were found between cohorts as both tmTNF and CD54 expressions by monocytes were comparable. Heterogeneity at the individual level was also visible. Made possible by the composition of the panel^10^, the engagement of NK cells through the ADCC MOA was also studied through the concomitant monitoring of both CD16 and CD69 on that particular cell subset. About 70% of naïve RA patients showed lower antibody-dependent cellular cytotoxicity (ADCC) ratio than healthy controls, thereby suggesting higher ADCC capabilities. However, statistically significant differences were not found between groups. Interestingly, PsA patients presented a significantly lower ratio in comparison to healthy donors as well. In contrast, treated RA patients did not show any response though ADCC mechanism. (Supplementary Fig. S4). Since 16 out of 17 treated patients had already been, or are currently, through at least one biological treatment, it could be hypothesized that the considered RA treated donors could be less responsive to IFX as a result of a previously mounted therapy resistance mechanisms. Still, no correlation nor distinction was found when looking at the donors who already have had an anti-TNF treatment, hence this hypothesis would need to be further studied. Though, these results are in agreement with previous studies, which reported a decreased production of TNF in already treated RA patients, either in the case of methotrexate or anti-TNFs treatments^42–44^.

## Conclusion

The proposed whole blood based functional assay could represent a valuable tool for RA patient characterization. In addition to demonstrating basal differences between naïve and healthy donors for the expression of some activation marker expression, dysregulated activation capabilities upon LPS stimulation have been reported. Sample clustering profiles have been shown to strongly depend on the activation condition, not only when basal and LPS conditions were compared but also when anti-TNF *in vitro* treatment was performed. Furthermore, different profiles within the naïve to treatment RA patient cohort were found, highlighting the importance of studying cell functionality to reveal patient stratification features. With this proof-of-concept study we aimed to demonstrate the relevance of whole blood based functional assays in translational studies. To continue evaluating the potential use of this approach in the context of response prediction, larger cohorts, as well as further exhaustion and activation markers should be considered. Moreover, the naïve-to-therapy patients considered here will be longitudinally monitored after therapy administration.

## Supplementary Information

Supplementary Information.

## Data Availability

All data generated or analysed during this study are included in this published article (and its supplementary information files).
