# Supplementary material for: Leveraging whole blood based functional flow cytometry assays to open new perspectives for rheumatoid arthritis translational research
Source: Sci Rep. 2022 Jul 16;12:12166. doi: 10.1038/s41598-022-16622-4 (PMC9288473; doi:10.1038/s41598-022-16622-4)
Supplement: Supplementary file 1 — Supplementary Information. [file 41598_2022_16622_MOESM1_ESM.docx]

**Supplementary information**

## **Table S1. Patient characteristics.** A Chi-square test was performed for the 5 first rows and statistically significant differences were considered when *p-values<0,05.

## **Table S2. Patient clinical scores.** DAS28 scores for RA patients and DAPSA scores for PsA patients.

**Figure S1. Gating strategy.** Leukocytes were first selected according to the CD45^+^ expression. Lymphocytes, monocytes and granulocytes were distinguished by CD14 expression and side scatter. Further lymphocyte distinction into NKCD56^bright^, NKCD56^dim^, NKT, and T cells was possible by CD56 and CD3 expression. Moreover, SSlow CD56^-^CD3^-^ cells were considered as mostly B cells.

**Figure S2. Unsupervised analysis of all parameters considered for the negative condition considering naïve RA and PsA patients.** (A) Response screen of basal Mean Fluorescent Intensities (MFIs) ranking all parameters according to its discriminative power to distinguish naïve RA from PsA patients. (B) PCA were only most discriminant features between groups were considered. (C) Box plots for CD69 on NK, NKT and CD62L on T cells MFI in naïve RA and PsA patients. Statistical significance was determined by a nonparametric Wilcoxon test, with *p-values < 0,05.

**Figure S3. Summary table of the observed differences between controls and Naïve RA patients in the basal and LPS conditions.** Raw MFI values were considered to represent differences in the basal conditions while stimulation indexes where considered in the LPS stimulation condition, representing the capabilities of each cohort to respond to LPS.

**Figure S4. ADCC ratio considering the healthy, naïve, treated RA and PsA patients after IFX treatment**. ADCC ratio was calculated with the percentage of CD69^+^ CD16^+^ NKdim cells divided by the percentage of CD69^+^ CD16^-^ NKdim cells.
